# Portable and Air Conditioner-Based Bio-Protection Devices to Prevent Airborne Infections in Acute and Long-Term Healthcare Facilities, Public Gathering Places, Public Transportation, and Similar Entities

**DOI:** 10.7759/cureus.55950

**Published:** 2024-03-11

**Authors:** Madhavan Pisharodi

**Affiliations:** 1 Neurosurgery, South Texas Neurosurgery Associates, Brownsville, USA

**Keywords:** covid-19, viral pathogens, airborne pathogens, immunology, healthcare economics, public health, infectious diseases, health care, microbiology, virology

## Abstract

The nature in which the coronavirus disease 2019 (COVID-19) pandemic started and spread all over the world has surprised and shocked experts and the general population alike. This has brought out a worldwide desire and serious efforts to prevent, or at least reduce, the severity of another airborne viral infection and protect individuals gathering for various reasons. Toward this main purpose, a novel method to disinfect the air, using graded, predictable, safe, and reliable dosage of ultraviolet C (UVC), with specially designed devices, is described here. Individuals exclusively breathing this disinfected air can prevent infection, thus destroying the airborne virus or any other pathogens outside the human body to prevent acute and chronic damage to the organs and provide a sense of security to congregate, use public transport, and be protected in acute and long-term healthcare facilities.

The study involved designing and testing a unit with one UVC chamber and another unit with six UVC chambers both enclosed in UVC-opaque housings that could be used to destroy airborne pathogens. Wild-type severe acute respiratory syndrome coronavirus 2 (SARS-CoV-2) was used as a representative pathogen. The virus was fed into these units and in both units, the virus was destroyed to undetectable levels. Such disinfected air can be made available for individuals to breathe at an individual and a community level.

The two units that were studied were able to destroy the SARS-CoV-2 virus completely in UVC-opaque housings, making them safe for human use. By employing the air to bring the virus to the UVC, the problem of the virus getting protected behind structures was avoided. The individuals get to breathe totally disinfected air through a mask or a ventilator. To protect individuals who are unable or unwilling to use these units meant for individual use, the same principle can be expanded for use with air conditioners to provide community protection. It is envisaged that this method can prevent airborne infections from turning into pandemics and is a clear example of advocating prevention, rather than treatment.

These units are expandable and the UVC dosage to the pathogen can be adjusted and predictable, thereby making it a standard technique to study the dosage needed to inactivate different pathogens.

## Introduction

The focus of this study is to make ultraviolet C (UVC) safe for regular use at an individual and community level by enclosing UVC in UVC-opaque housings and making sure that the individuals in both situations are not exposed to UVC in any way. The system uses air to take the pathogen to the UVC for its total and safe destruction. The added benefit is the ability to destroy the pathogen totally outside the body, thus avoiding the actual invasion of the body by the pathogen, which could cause multi-organ pathologies and multiple disabling symptoms from acute infection, cytokine storm, or long COVID-like delayed complications. The ability to reduce the need for extensive lockdowns has unimaginable social, financial, political, and other benefits. Some measures that can possibly prevent the next outbreak of airborne infection going into a pandemic are also mentioned.

Global impact

The 11th of March 2023 marked the 3rd anniversary of the day the World Health Organization (WHO) characterized the infection from SARS-CoV-2 (COVID-19) as a pandemic. During the last three years of the pandemic, most people have had family members or friends who died from COVID-19, some of them in an undignified tragic fashion reminiscent of the days of the black death caused by *Yersinia pestis*. Even though science was able to analyze why SARS-CoV-2 behaved so differently from SARS-CoV-1 of 2002-2004 and brought humanity to its knees by threatening our health and financial stability, it has not provided a viable plan to abort another airborne pandemic in the future. The global financial loss from this pandemic in terms of reduced gross domestic product (GDP), lost working man-hours, and reduced potential for future growth is difficult to calculate in real numbers but is estimated to be the worst since the great depression. In 2020, the United Nations predicted “this marks the sharpest contraction since the great depression in the 1930s” [1,2]. The catastrophic effect on our health includes not only the deaths and sufferings from the acute illness but also the now evolving observations of delayed harm to the body in the form of long COVID.

COVID-19 has so far turned out to be a major disaster for the world in the 21st century and more so for the USA. The deaths in the USA were significantly high from the very beginning. By the 25th of May 2020, there were more than five million cases worldwide, most of them (1.6 million) in the USA [3]. More recently, the WHO estimated the deaths in the USA at about 16% of total reported deaths in the world (i.e., 1,117,054 out of 6,887,000). This is significantly more than the 1.35% (675,000 out of 50 million) deaths in the USA from the 1918 flu pandemic. It is important to find out the reason for this change and we should also look for ways to prevent this from happening in the future.

Unrecognized by many people, there is a possibility that the air conditioning gave some protection for the virus viability and facilitated airborne infection. The conditioned dry air from our conventional air conditioners (ACs) gets colder and more stagnant the further we go down from the ceiling, especially in cold climates, because the warm air tends to go up in the room. When the AC fan is on “Auto” mode and turns off for a certain amount of time every day, the air is absolutely still. People usually move around in this cold stagnant air in the lower part of the room exposing themselves to the virus for a long period. It is possible that this lack of movement of air in air-conditioned facilities and homes contributes to the “flu season” also. Flu viruses have caused several pandemics, including the mother of all pandemics in the years 1918 and 1919. This pandemic infected about 500 million or 33% of the world population at that time and caused 50 million deaths worldwide, of which 650,000 were in the USA [4]. Record documents at least three pandemics before the 1918-1919 pandemic and three afterward: 1957-1958, 1968-69, and 2009-2010. The multiple variants and the easiness of their mutations keep the flu viruses coming back each winter, necessitating the formulation of new vaccines almost every year. In the tropics, influenza shows no clear seasonality. It is estimated that seasonal influenza kills about 650,000 people globally and 36,000 in the United States every year. There is a possibility that present-day air conditioning systems allow the flu viruses to infect people, mutate, and develop variants in a regular relay fashion every year during the cold months. It is also possible that we can break the cycle by adopting the systems described here.

The reality of long COVID pathologies, and the possibility that the new variants can evade the vaccines and antivirals, make it a better option to prevent the virus from entering the human body rather than fighting the virus inside the body. The technique described here has the potential to prevent the virus from entering the body. This can be accomplished by destroying the virus in the air we breathe by using UVC in a safe and reliable way. This should be done for an individual as well as for the whole community. Individuals can be protected by using portable units and the community should be protected by some modifications to the present-day ACs. Today, even developing countries have ACs for hospitals and other healthcare facilities. There is a high probability that these air-conditioned buildings are the locations where the virus is infecting more and more individuals. We can use the same air-conditioned facilities, with some modifications, to limit the spread of the virus. With these arrangements, if individuals and air-conditioned buildings are equipped with methods to kill these viruses, rather than blocking and allowing them to recirculate, the airborne outbreaks of infections can be contained at the source. Masks, social distancing, and lockdown are methods to avoid the virus and not to destroy it. The units described here can destroy the virus and potentially prevent the virus from entering the human body.

The bats have already given us severe acute respiratory syndrome coronavirus (SARS-CoV), Middle East respiratory syndrome-related coronavirus (MERS-CoV), and SARS-CoV-2 pandemics in the 21st century. The frequency of these zoonoses, accelerated by increased human intrusion into previously undisturbed habitats, suggests that new coronaviruses and other emerging pathogens will continue to threaten human health [5]. Such viruses can cause diseases in human, animal, and plant systems [6]. Airborne and waterborne pathogenic viruses are among the most important global risks faced by mankind [7]. Inactivation of the viruses is one of the safest goals to prevent the spread of the infection [7]. Virus inactivation can be accomplished (outside the human body) using physical and chemical methods [7]. Chemical inactivation can be done by using detergents, solvents, acidic pH, ethanol, and sodium hypochlorite [8,9]. Ultraviolet rays in the C range of wavelength (UVC) have a lethal effect on SARS-CoV-2 and similar pathogens. The use of UVC is severely restricted by the WHO, FDA, and CDC because of its potential to cause skin and eye damage and cancers. UVC being an invisible light ray can be blocked by objects in the way and the virus can hide behind them. By avoiding both these drawbacks the devices presented here can be made safe, reliable, and effective for the destruction of the virus and many other pathogens outside the human body. Using UVC to disinfect water is an evolving science with great potential for controlling waterborne infections. The techniques described here have great promise to prevent airborne infections.

There are still many outstanding questions (relating to this pandemic) whose answers may assist in the development of new tools to control these viruses [5]. While hoping that COVID-19 will slowly wither away and another similar one will never come again, we must be prepared. Any attempt to control an airborne infection starts with the understanding of the special features of the virus and the stages of infection itself.

Lessons from the COVID-19 pandemic

To date, no definite therapeutic agents are available against COVID-19 [10]. The severity of COVID-19 is linked to its cytopathic effects and the escape of the virus from the host immune system [5]. Looking back on the behavior of COVID-19, and the SARS-CoV-2 that caused it in the last three years, there are some observations that can help us in the future. Once SARS-CoV-2 enters our body, it can manipulate the function of our cells to establish infection and determine its severity. The ability of SARS-COV-2 to spread, from asymptomatic and pre-symptomatic infected individuals, is a major factor in the rapid spread of this pandemic. Even for the individuals developing symptoms, they can be infectious two to five days before the first symptoms manifest [3]. It is estimated that 30% of the infections are asymptomatic [11] and more than 50% of the transmission occurs from asymptomatic individuals or from the pre-symptomatic phase [12]. The significance of this is underestimated. For the same reason, it was a challenge to estimate the real R-naught (Ro) for COVID-19. All these made isolating the sources of infection, and quarantining the infected individuals, difficult and unreliable [13].

By spreading the infection like wildfire silently and misleading us with a low mortality rate (2.1% compared to 9.6% for severe acute respiratory syndrome (SARS) and 34.4% for Middle East respiratory syndrome (MERS)), the virus managed to propagate in an undetected way in humans [3]. COVID-19 presented a low level of warning in its early days until the pandemic extended itself into an irreversible stage of wild global presence at the start of the pandemic and at the start of every new variant of concern. Such worldwide existence of infected individuals provided a wide choice for the virus to mutate and come up with new variants, which are, usually, more infectious [14]. Immunocompromised individuals and individuals with high comorbidity, including patients on dialysis, are better choices for the virus to mutate and create variants [15]. Any mutation that improves the infectivity of the virus creates many progenies of the virus with that mutation, and the virus equipped with this mutation turns into a new variant. When viruses evolve to improve immune surveillance for their survival, they often suffer reduced fitness and become less severe [16-18]. Rapid evolution and new variants of SARS-CoV-2 with higher transmission rates and variable lethality continue to appear in circulation every few months [14]. Since reduced severity with the new variants does not happen every time, a more infectious, more lethal variant can come up at any time in the future.

The most effective way to reduce mutations and the development of new variants is obviously by reducing the total number of infected individuals in the community. Disinfecting the air we breathe will reduce infections, mutations, and new variants by eliminating the virus before it gets into the human body.

One of the clear examples of underestimating the seriousness of the problem in the early days of the pandemic was the hesitation to accept the possibility that the virus may be airborne and can spread through the air. While the diameter of SARS-CoV-2 is about 0.1 μm (micrometer) to 0.5 μm, that of a smoke particle is about 0.4 μm to 0.7 μm [19]. This shows that naked virus particles are smaller than smoke particles and can possibly create virus smoke in a room without cross-ventilation, as in our air-conditioned rooms. Except in situations like mouth-to-mouth resuscitation, the virus must travel through the air, whether it is in the form of droplets or aerosols, and even for most of the fomite transmission. Clearing the air around a virus spreader completely, without harming the spreader, is an absolute, basic, necessity to prevent the spread of the infection.

Unlike the SARS-CoV, which started in 2002 and ended in 2004, the lightning spread and the defense mechanisms of SARS-CoV-2 made the prevention of the present pandemic difficult despite all the efforts to contain the initial infection. Overcrowding on a global scale, fast and efficient travel for businesses, pleasures, social, or other reasons, indiscriminate indoor crowding, and a sense of individual freedom are all factors that contributed to this pandemic in addition to the characteristics of the virus itself. The increased affinity of SARS-CoV-2 receptor-binding domain (RBD) for angiotensin-converting enzyme 2 (ACE2), compared to SARS, may account for the significant airborne transmission of SARS-CoV-2 [20]. The newer variants have in general come up with increased affinity for ACE2. These variants create a situation of "moving targets" for our vaccines and antivirals, providing the new variants enough time to establish a global presence before our vaccines and antivirals are reprocessed to catch up.

Hospital-acquired infections (HAI)

HAI can come from acute or long-term healthcare facilities, healthcare workers, and other entities providing healthcare to individuals. These can come from physical or fomite contacts, airborne or equipment-related, including infections like ventilator-associated pneumonia. It is reported that about one-third of COVID-19 deaths were in long-term healthcare facilities. It is hoped that through the use of portable, personal bio-protection devices (PPBD) and modified and disinfected ACs, this major problem can be controlled. The amount of lives saved and expenses averted will be unimaginable. PPBD and ventilators will give excellent protection to an individual. The community protection through modified disinfected ACs needs some explanation. If a hospital is assumed to have rooms A, B, and C, and if there is a pathogen spreader in room A, rooms B and C will be protected very well and room A is only protected partially. The partial protection is by avoiding the build-up of virus pathogen smoke in the room and real-time clearance of the pathogen. Masks and social distancing will be desirable in room A if the individual is not using PPBD. Also, there has to be a way of air washing clothing, masks, PPBD, etc. when one leaves such a room. With some planning, this system can be built to provide excellent protection in healthcare facilities and significantly reduce HAI.

## Materials and methods

We have tested two UVC units designed and created in our facility. Both units are specifically addressing aerosolized microorganisms, and in the testing, we used airborne SARS-CoV-2 virus to ensure that the research is technically sound. Adequate level 3 lab protection was used for the testing.

Virus and TCID50 assay

SARS-CoV-2, strain USA _WA1/2020, was used for the study. SARS-CoV-2 virus suspensions were prepared on the day of bioaerosol runs from frozen seed stock initially generated (one passage) in Vero C1008 (E6) cells (BEI Resources, NR-596, Lot 3956593) from lyophilized material provided by the World Reference Center for Emerging Viruses and Arboviruses at the University of Texas Medical Branch (TVP 23156). Next-generation sequencing confirmed a 100% consensus sequence-level match to the original patient specimen (GenBank accession MN985325.1) to make sure that the virus used is authentic.

One day prior to assay execution, a well-characterized, low-passage Vero E6 cell bank (originating from BEI Resources NR-596) was used to seed 96-well culture plates at 2 x 10⁴ cells per well. Cells were incubated under optimal conditions (37°C/5% CO₂) for 16-24 hours or until greater than 90% cell confluence was achieved. On the day of testing, each sample collected during the aerosol tests was serially diluted (10-fold) in a dilution medium (minimal essential medium/2% heat-inactivated fetal bovine serum (HI-FBS)). Sample dilutions were added to replicate wells (0.1 mL per well, five wells per dilution). For each 96-well plate, positive virus-only control and negative media control were incubated in parallel to ensure that the test and control studies were used at every possible stage. Cultures were incubated under optimal conditions for SARS-CoV-2 (37°C/5% CO₂) for 72 hours, after which cytopathic effect (CPE) was observed microscopically. Results were tabulated and used to calculate the 50% tissue culture infectious dose (TCID50). The lower limit of detection (LLOD) for the assay was 63.2 TCID50/ml or 1.8 logs.

Aerosol generation and sampling

**Figure 1:**
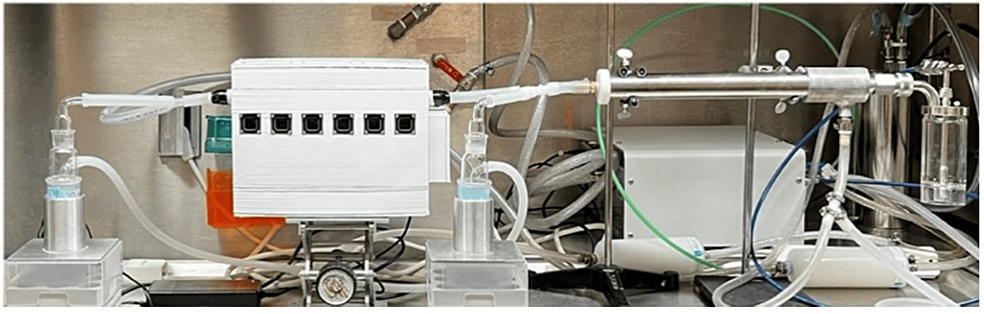
Aerosol testing setup Large unit setup at the facility of the Galveston National Laboratory.

The aerosolization of the infectious agents was accomplished using an automated aerosol control platform (Biaera Aero3G; Biaera Technologies, LLC, Hagerstown, MD). The bioaerosols were generated using a six-jet Collison nebulizer, which typically produces droplet sizes of approximately 1 μm. The total airflow rate to the test devices, comprised of both nebulizer air and diluter air, was 30 liters per minute (LPM). The nebulizer airflow rate to the six-jet Collison nebulizer was 14 LPM. Bioaerosol samples were collected before and after the test devices for each aerosol run using SKC BioSamplers (SKC, Inc., Seoul, South Korea). The airflow rate to each BioSampler was approximately 19 LPM. The same sample airflow rate of 19 LPM was used for the six-chamber test, six-chamber control, one-chamber test, and one-chamber control. Aerosolization and bioaerosol sampling were performed for 10 minutes. All aerosolization procedures were performed in a class III biosafety cabinet housed with the animal biosafety level 3 (ABSL-3) facility of the Galveston National Laboratory. The aerosol setup is shown in Figure 1 (only a large test device is shown).

Test devices and study design

The fundamental units of testing are the UVC chambers. In the larger unit, there are six chambers and in the smaller unit, there is only one chamber. Since the variables were well controlled, the only factor that resulted in the difference in the UVC dosage was the number of chambers. This made the system very predictable and reliable unlike making UVC thrown on petri dishes over open air. The large unit was designed to be used with an AC unit. The smaller units were designed as a PPBD that can be used by healthcare workers and individuals otherwise exposed to pathogenic microorganisms up close. A review of the literature showed that this is the first time such a portable UVC unit has been created, enclosed in a UVC-opaque housing, and tested. It can also be incorporated into a ventilator or anesthesia machine to prevent the virus particles from contaminating the equipment and also to prevent the virus from going back to the patients. This can protect the patients and prevent hospital-acquired infections.

**Figure 2:**
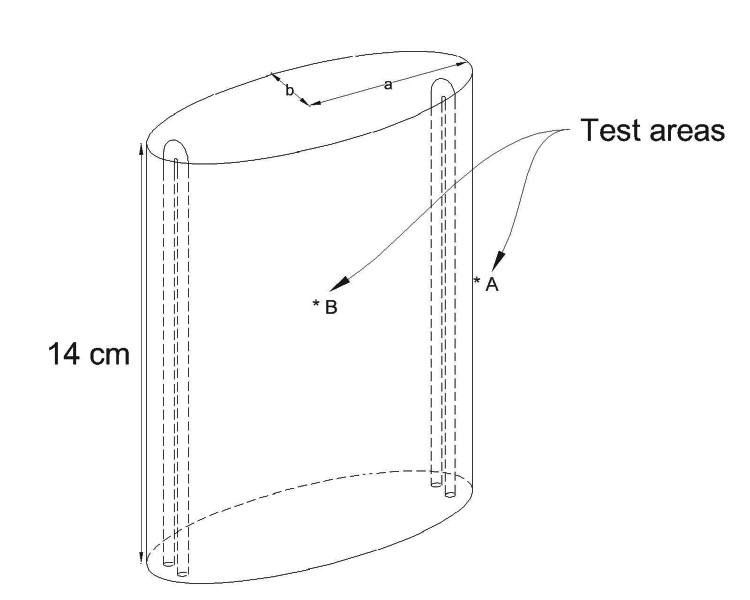
The oval chamber without helical air flow diverter measured ultraviolet (UV) intensity (A = 0.30 cm & B = 4.6 cm) from source: a = 10.16/2 = 5.08 cm; b = 3.81/2 = 1.91 cm Image credit: Madhavan Pisharodi, MD.

As shown in Figure 2, each chamber has a height of about 14 cm, and it was the same for one chamber in the one-chamber unit and six chambers in the six-chamber unit. The chambers are oval with 10.16 cm x 3.81 cm dimensions. The circumference of the chambers was calculated as 21.93 cm. Through a special arrangement, the air in the chamber is made to circulate around the circumference five times from one end to the other, making a passage of about 109.77 cm per chamber, and this is the passage in the one-chamber unit. In the six-chamber unit, this will be around 655.32 cm, which is 109.77 times 6. This means that the variables are only the number of chambers, nothing else. There are two U-shaped UVC lamps on the narrow sides of each oval chamber, creating four vertical UVC tubes per chamber. We used a General Tools (Secaucus, NJ) Digital Ultraviolet UVC light meter (220-270 nm) to measure the UVC intensity. We measured the intensity at 0.3 cm and 4.6 cm from the UVC source and the numbers were 19.14 mW/cm² and 5.04 mW/cm², respectively, as shown in Table 1. We realized that there is no shadow or structure preventing the air from being in direct contact with the UVC source and the 0.3 centimeters is not the minimum distance from the UVC lamp to the air loaded with the microorganisms, but for calculation purposes, we will use these numbers. It is exactly the same measurements in the one-chamber unit and the six-chamber unit. We calculated the average intensity of UVC inside the chamber as 24.18 (19.14 + 5.04) divided by 2 and came up with 12.09 mW/cm².

**Table 1:** The recorded UV intensity values when measuring in different spots in a chamber UV: ultraviolet.

2 lamps per chamber (1 chamber)	Point B at 4.6 cm from the lamp	Point A at 0.3 cm from the lamp	Average UV intensity
Test chamber: Aluminum spiral, aluminum wall	Lowest: 5.04 mW/cm^2^	Highest: 19.14 mW/cm^2^	Average: 12.09 mW/cm^2^

The calculated airflow rate to these units for the test was 19 LPM (19,000 cm³). If an adult breathes 12 per minute and the average air intake is about 500 cc, the total air intake will be about 6 liters (6000 cm³) per minute. The velocity of the 19,000 cm³ per minute flow through the chamber is calculated by dividing the airflow by the area of the chamber. The area of the chamber is calculated to be pi x (a * b) = 3.14 x (5.08 cm * 1.91 cm) = 30.38 cm². This is the area of the oval chamber at any cross-section. The resulting velocity was calculated by dividing the airflow by the area. For 19,000 cm³ per minute, this was 625.4 (19,000/30.38) cm per minute or 10.42 cm per second. Since the combined length of six chambers is 84 (14 x 6) cm, the time taken for the airflow through the six chambers (without the helix) at 19,000 cm³ per minute is about eight seconds by dividing 84 cm by 10.42 cm/s. If we reduce the airflow to 6000 cm³ per minute (as in normal breathing), the time taken for the air travel in the unit will be about 25.3 seconds. The corresponding numbers for the single chamber unit are 1.33 seconds and 4.2 seconds in a chamber without the helix.

Each chamber has a five-disc helical device with an area of 15.68 cm² per disc. The air in the chamber has to navigate the space between the chamber walls (30.38 cm²) and each of the helical devices takes up an area of 15.68 cm², which measures around 14.707 cm². The air will be subjected to a centrifugal force pushing the particles in the air close to the periphery of the cylinder. This will result in the particles in the air having to travel about 109.77 cm in each chamber (5 x Sqrt (21.93² + 1²)) and is the same whether it is in the one-chamber unit or in the six-chamber unit where the distance has to be multiplied by six.

The velocity of the air will increase in the presence of a helix because of the reduced area. The new velocity will be 19,000 cm³/per minute divided by 14.707 cm² and will be 1,291.9 cm per minute or 21.5 cm/s. Since the length of the air travel is 109.77 cm, the time taken for the air to travel one chamber will be 109.77 cm divided by 21.5 cm/s and will come to about 5.1 seconds. If the airflow is at 6 liters per minute, the time taken per chamber will be about 16.04 seconds. The corresponding numbers for the six-chamber units will be 30.46 seconds and 96.26 seconds, respectively as shown in Table 2.

**Table 2:** The time it will take for the air to pass through one and six chambers at different LPM with and without helix LPM: liters per minute.

	Air flow passage without helix		Air flow passage with helix	
	1 chamber, length: 14 cm	6 chambers, length: 84 cm	1 chamber, length: 109.22 cm	6 chambers, length: 655.32 cm
Avg. breathing rate: 6 LPM	4.2 seconds	25.3 seconds	16.04 seconds	96.26 seconds
Consistent: 19 LPM	1.33 seconds	8 seconds	5.1 seconds	30.46 seconds

The lowest measured intensity of UVC inside the chamber was 5.04 mW/cm² and the highest was 19.14 mW/cm², which brings the average to be at least 12.09 mW/cm² without taking into account that there is no 0.3 cm gap between the UVC source and the air. This is the same for the one-chamber unit and for each of the chambers in the six-chamber unit (Table 1). Since the UVC dose is the intensity multiplied by duration [21], the minimum UVC dose at 19,000 cm³ per minute (19 LPM) for the two units will be 25.7 mJ/cm² (5.04 mW/cm² x 5.1 sec) and 153.51 mJ/cm² (5.04 mW/cm² x 30.46 sec) and the maximum for the two units will be 97.6 mJ/cm² (19.14 mW/cm² x 5.1 sec) and 583 mJ/cm² (19.14 mW/cm² x 30.46 sec). At 6000 cm³ per minute (6 LPM), the corresponding numbers will be 80.84 mJ/cm² (5.04 mW/cm² x 16.04 sec) and 485.2 mJ/cm² (5.04 mW/cm² x 96.26 sec) as the minimums and 307 mJ/cm² (19.14 mW/cm² x 16.04 sec) and 1842.4 mJ/cm² (19.14 mW/cm² x 96.26 sec) as the maximums for the two units (Table 3). The average for one chamber with the helix for 19 LPM is 61.66 mJ/cm² and for 6 LPM is 193.9 mJ/cm². The average for six chambers with the helix for 19 LPM is 368.23 mJ/cm² and for 6 LPM is 1163.78 mJ/cm². These numbers are extremely high compared to the published UVC doses required to inactivate the SARS-CoV-2 virus (Table 4). It is possible that these units can be reduced in size after appropriate quantitative studies. These sample sizes are constant and predictable, unlike the previously available techniques of UVC treatments.

**Table 3:** UV doses at different liters per minute in a one-chamber & six-chamber device with a helix UV: ultraviolet.

	1 chamber		6 chambers	
	6 LPM, 16.04 sec	19 LPM, 5.1 sec	6 LPM, 96.26 sec	19 LPM, 30.46 sec
Minimum UV dose	5.04 mW/cm^2^ x 16.04 sec = 80.84 mJ/cm^2^	5.04 mW/cm^2^ x 5.1 sec = 25.7 mJ/cm^2^	5.04 mW/cm^2^ x 96.26 sec = 485.2 mJ/cm^2^	5.04 mW/cm^2^ x 30.46 sec = 153.51 mJ/cm^2^
Maximum UV dose	19.14 mW/cm^2^ x 16.04 sec = 307 mJ/cm^2^	19.14 mW/cm^2^ x 5.1 sec = 97.6 mJ/cm^2^	19.14 mW/cm^2^ x 96.26 sec = 1842.4 mJ/cm^2^	19.14 mW/cm^2^ x 30.46 sec = 583 mJ/cm^2^
Average UV dose	12.09 mW/cm^2^ x 16.04 sec = 193.9 mJ/cm^2^	12.09 mW/cm^2^ x 5.1 sec = 61.66 mJ/cm^2^	12.09 mW/cm^2^ x 96.26 sec = 1163.78 mJ/cm^2^	12.09 mW/cm^2^ x 30.46 sec = 368.26 mJ/cm^2^

**Table 4:** UV doses required to inactivate SARS-CoV-2 according to research UV: ultraviolet.

UV dose (mJ/cm^2^) at 254 nm	Log inactivation	References
16.9	Log 6 (99.9999%)	[22]
1.5	Log 2 (99%)	[23]

The six-chamber unit (large) and the one-chamber unit (small) were tested. The large unit was plugged into a 110V outlet within the chamber, while the small unit was powered by an internal battery pack. For the experimental runs during which the devices were turned on, the devices were allowed to warm up for five minutes prior to aerosol generation. For the control runs, the devices remained off. The study design is shown in (Table 5). This way the control and test devices had the exact variables except for the UVC being on and off.

**Table 5:** Study design

Day 1			
Device	Device setting (on/off)	Number of aerosol runs	Viral agent
Large purifier	On (experimental)	3	SARS-CoV-2
Large purifier	Off (control)	3	SARS-CoV-2
Day 2			
Device	Device setting (on/off)	Number of aerosol runs	Viral agent
Small ventilator	On (experimental)	3	SARS-CoV-2
Small ventilator	Off (control)	3	SARS-CoV-2

## Results

Tables 6, 7 provide the viral concentrations of the samples collected from the aerosol samplers during the experimental (devices on) and control runs (devices off) when testing the large and small devices, respectively. There were a total of 24 samples. There were 12 for the six-chamber and 12 for the one-chamber unit. The 12 samples included pre-unit and post-unit for the three tests and three controls for each unit. The pre-unit sampler refers to the in-line aerosol sampler placed after the test device. For both the large and small test devices, the virus could not be detected in the post-unit samplers during the experimental runs (when the devices were on), which indicates that all aerosolized virus was inactivated. However, the LLOD for the TCID50 assay was 63.2 TCID50/ml (or 1.80 logs); therefore, the actual viral concentration for the experimental runs could be anywhere from 0 TCID50/ml to just below 63.2 TCID50/ml. On the contrary, the virus was detected in the samples collected from the post-unit aerosol samplers during the control runs (when the devices were off) for both devices.

**Table 6:** Viral concentration in aerosol samplers using the large test device * The number in parentheses is the log-transformed value of viral concentration.

Experimental runs			Control runs		
Run	Pre-unit sampler concentration TCID50/ml	Post-unit sampler concentration TCID50/ml	Run	Pre-unit sampler concentration TCID50/ml	Post-unit sampler concentration TCID50/ml
1	3.98 x 10^4^ (4.60)*	<6.32 x 10^1^ (1.80)*	4	8.41 x 10^3^ (3.92)*	4.92 x 10^3^ (3.69)*
2	3.98 x 10^4^ (4.60)*	<6.32 x 10^1^ (1.80)*	5	1.53 x 10^4^ (4.18)*	1.08 x 10^4^ (4.03)*
3	7.84 x 10^3^ (3.89)*	<6.32 x 10^1^ (1.80)*	6	9.45 x 10^3^ (3.98)*	3.69 x 10^3^ (3.57)*

**Table 7:** Viral concentration in aerosol samplers using the small test device * The number in parentheses is the log-transformed value of viral concentration.

Experimental runs			Control runs		
Run	Pre-unit sampler concentration TCID50/ml	Post-unit sampler concentration TCID50/ml	Run	Pre-unit sampler concentration TCID50/ml	Post-unit sampler concentration TCID50/ml
1	7.11 x 10^3^ (3.85)*	<6.32 x 10^1^ (1.80)*	4	1.77 x 10^4^ (4.25)*	6.22 x 10^3^ (3.79)*
2	4.59 x 10^3^ (3.66)*	<6.32 x 10^1^ (1.80)*	5	3.16 x 10^4^ (4.50)*	2.31 x 10^4^ (4.36)*
3	1.47 x 10^4^ (4.17)*	<6.32 x 10^1^ (1.80)*	6	9.74 x 10^4^ (4.99)*	3.69 x 10^4^ (4.57)*

Tables 8, 9 summarize the reductions in the log viral concentration of the samples collected from the aerosol samplers during the experimental (devices on) and control runs (devices off) when testing the large and small devices, respectively. The reduction in log viral concentration was calculated by taking the difference between the log viral concentration of the samples collected from the post-unit sampler and the log viral concentration of the samples collected from the pre-unit sampler (Tables 6-9). The net log reduction is the log reduction of the experimental runs minus the log reduction of the control runs. The average reduction in the log viral concentration for the experimental runs using the large device was >2.56 logs, which corresponds to >99.7%. This was the maximum degree of inactivation that could be assessed due to the LLOD of the quantitation assay. For the control runs, there was also a decrease in log viral concentration, but it was small (0.26) relative to the experimental runs. The average net log reduction was >2.30 (2.56-0.36) logs, which corresponds to >99.5%. For the small device, the average reduction in the log viral concentration for the experimental runs was >2.09 logs, which corresponds to >99.2%. Once again, this was the maximum degree of inactivation that could be assessed due to the LLOD of the quantitation assay. The average net log reduction (after accounting for the average reduction measured for the control runs) was >1.75 (2.09-0.34) logs, which corresponds to >98.2%. The log determination and the calculations of the difference between the test and control were done using the usual statistical calculations.

**Table 8:** Log reduction in viral concentration using the large test device

Device setting	Run	Log reduction	Device setting	Run	Log reduction	Net log reduction
On (experimental)	1	>2.80	Off (control)	4	0.23	>2.57
	2	>2.80		5	0.15	>2.65
	3	>2.09		6	0.41	>1.68
Averages		>2.56			0.26	>2.30

**Table 9:** Log reduction in viral concentration using the small test device

Device setting	Run	Log reduction	Device setting	Run	Log reduction	Net log reduction
On (experimental)	1	>2.05	Off (control)	4	0.46	>1.59
	2	>1.86		5	0.14	>1.72
	3	>2.37		6	0.42	>1.95
Averages		>2.09			0.34	>1.75

The two test devices inactivated airborne SARS-CoV-2 to undetectable levels. The results show that the net percent reductions in viral concentration achieved by the large and small devices were >99.7% and >99.2%, respectively; however, since no virus was detected in the samples collected from the post-unit aerosol samplers, and accounting for the LLOD of the viral quantitation assay, the true percent reduction likely exceeded these percentages. Overall, the devices showed maximum inactivation efficiency using this test system. The differences between the test and control were statistically significant.

## Discussion

Since we know by now that the SARS-CoV-2 virus has some very dangerous qualities, we must seriously plan how to tackle this virus, and other such microorganisms coming up in the future. One simple principle is to inactivate such organisms outside the body and prevent acute and chronic pathological sequelae in the body caused by the infection, the cytokine storm, DNA alterations, and many other related pathologies. The devices described here are planned to totally disinfect the air we breathe. There are no such devices that can disinfect the air completely as described here. There are three types of UVC devices available today. The first one is like ceiling lights that can throw UVC down and disinfect the surface of structures below. This has very limited use. For example, if there are three layers of instruments, this can disinfect only the top surface of the top layer. The second type is the free-standing UVC units that take in the ambient air, disinfect, and put out the disinfected air. This is almost like a table fan and protects only the air just in front of it. The third type is even less useful because it is in the form of boxes to disinfect phones, pens, etc.

In comparison, the PPBD presented here has two parts working in conjunction to give totally disinfected air as the exclusive breathing air for the individual. The UVC unit is a housing that is opaque to UVC and forces the incoming virus to be in very close contact with the UVC source for a desired length of time for complete destruction of the virus. The disinfected air is then carried through a tube to a second unit, which is a barrier mask that has no pores and gives an airtight fitting over the face. This makes sure that the individual can be standing next to a COVID-19 patient, coughing or sneezing out the virus, and still the virus cannot get into the individual. For further details, see the patents [24-26] and the publications [27,28].

Similarly, a system for disinfecting the entire room or the whole building by incorporating a UVC housing in line with the AC is also new and has never been done before. This is also described in the same patents [24-26] and publications [27,28].

We can do this by utilizing UVC of either 254 nm frequency or 222 nm. Studies have confirmed that a wavelength of 254 nm is considered the most effective for the germicidal activity of viruses [29]. Some studies have suggested that UVC of 222 nm is equally or more effective against SARS-COV-2 and that it is less damaging to the skin. However, UVC of 222 nm is known to produce ozone, which can cause some additional risks, including increased mortality (FDA safety communication, February 27, 2020, Environmental Protection Agency (EPA), USA - July 20, 2022). Even though Far UVC 222 is considered safer for the skin, it can also produce skin damage when the exposure exceeds 50 millijoules per centimeter square [30]. The risk of ozone causing asthma-like symptoms with or without damage to the respiratory tract most likely outweighs the benefits of the ozone having a lethal effect on microorganisms. Far UVC 222 is also more difficult and expensive to produce. Encouraged by the notion that Far UVC 222 is safer than UVC 254, indiscriminate use of UVC 222 without proper warning and protection is counterproductive.

A 16 cm long single-chamber unit and a 32 cm long six-chamber unit were tested. SARS-CoV-2 (wild type) virus was used for our experiments and total inactivation was achieved using UVC of 254 nm wavelength (Tables 6, 7). Control studies using the same variables were used to make sure that the observed inactivation was clearly from UVC radiation. Six test and six control samples were passed through specifically created UVC chambers where the air was made to navigate long distances in close contact with UVC. The UVC lamps were turned on in all the test series, and in the control ones, the UVC lamps were turned off. The UVC lamps were inside the chambers, and these were enclosed in UVC opaque housings. The unique way of arranging the helical rungs in the chamber made the air go through a serpentine pathway and the distance traveled by the air in one chamber was about 109.77 cm long and in the six-chamber unit, the air traveled inside the housing for about 655.32 cm. In both units, with all the six passes, the SARS-CoV-2 was destroyed to undetectable levels. By using such a unit from remote housings, totally disinfected air can be provided to an individual through air-tight masks and/or ventilators and to a building through a modified air-conditioning system, without exposing the end users to the UVC light. By making the circulating air mop up the viruses from under the tables and behind the shelves, the problem of the viruses hiding from the UVC is eliminated. The calculated UVC dosage ranged from 25.7 mJ/cm² to 1842 mJ/cm² between the two units under a variety of settings. Unlike other previous UVC applications, the dosage in these units is quite predictable.

The dosage and quality of UVC required to destroy the different types of bacteria, viruses, and other pathogens are different. Some of these dosage differences are due to the different testing methods presently in use. Several common pitfalls hinder accurate measurements of UVC wavelength and dose [31]. At present, no information is available to define the action spectrum of SARS-CoV-2 [32]. The development of an action spectrum for this virus will represent an important contribution to the effort to control the virus, especially in indoor settings. A predictable, reliable UVC dosage as can be provided with PPBD can be a benchmark set up for studying pathogens in the laboratory. “Properly designed experiments to measure the UVC dose-response behavior involve the use of a UVC exposure device that delivers a controllable, quantifiable dose to a viral population” [33]. “For airborne and surface-associated viruses, no such standard currently exists” [33]. The expandable nature, the controllable airflow amount, the controllable airflow velocity, and the standardized intensity of the UVC source make the devices presented here potential candidates for this much-needed system. Some of the common undesirable variables are eliminated in this system. The expandable nature of the arrangements in the units can benefit in many ways.

Death and organ damage from COVID-19

At the beginning of the COVID-19 pandemic, the death rate was projected to be around 2 to 3%. After three years, the WHO documents show that the reported global COVID-19 death rate was about 0.9%. About 30% of the infected individuals were asymptomatic while the total mild cases reported were about 80% of the symptomatic patients [34]. These numbers show that the human body has a strong defense mechanism fighting off potential pathogens, including airborne viruses. However, the long-term effects after a presumed cure of the infection are especially significant and alarming after some viral infections.

In the case of COVID-19, the viruses get into the human body mostly through the nose, the mouth, and the eyes; the viruses are cleared in most cases by the innate immune system through recognition, signaling, and elimination. The innate system then communicates with the adaptive system to clear the remaining viruses and create a memory for future reference.

Our experience in the last three years showed that vaccines can reduce the severity of illness but cannot prevent infections, reduce mutations, or prevent the development of new variants. The new variants are known to increase infectivity and possibly reduce the severity of the disease. It is believed that vaccines do not always control the infection rate but can reduce the severity. The interplay of the vaccines and the development of new variants regarding the question of the proportion of reduced severity from the two need to be critically evaluated.

Yet another factor to consider is the permanent damage done to humans with these viral infections. More so with viral infection than with bacterial, and other pathogens, the potential for such chronic damage done by viruses is by now obvious. While the other pathogens are mostly temporary intruders into our bodies, some of these viruses behave like they are our unwelcome but permanent residents. For instance, Parkinson’s disease (PD) has been observed to result after influenza, herpes simplex, and hepatitis B and C infections, and it may be due to permanent damage to the neurons. It has been reported that there is a 70% or higher risk of PD for those who had flu 10 or more years earlier, and a 90% for those who had flu 15 or more years earlier. The takeaway message is that these viruses continue to damage the structure and function of human cells for years after infection, some of them through their effect on our DNA, as seen in infections by the retroviruses. The now-evolving picture of chronic COVID-19 syndromes is rather depressing if not outright scary. The differentiation between long COVID syndrome (LCS), post-COVID syndrome (PCS), “chronic COVID-19,” “ongoing COVID-19,” “post-acute COVID-19,” and many others is arbitrary and mostly based on the duration of symptoms [35]. The sad fact is that PCS may develop even in patients with mild-moderate COVID-19 and even in asymptomatic patients [36,37]. It has been shown that PCS may progress in association with the development of mast cell activation syndrome (MCAS) [38]. In a survey of 136 long COVID-19 patients with 136 controls and 81 patients with MCAS, it was shown that the mast cell activation symptoms were increased in long COVID-19 and that the symptom profiles of long COVID-19 mimicked those of MCAS [39,40]. The roles of mast cells in SARS-CoV-2-induced hyperinflammation and cytokine storms have recently been one of the hot topics in the literature [41]. Prolonged signs and symptoms could be evident for seven to 15 years following previous SARS [42], and therefore, PCS may persist for years following COVID-19 [35]. In some patients with PCS, there is a counter-balanced anti-inflammatory response after the cytokine release syndrome (CRS) of acute illness [43,44], resulting in a prolonged immunosuppression leading to the “propagation catabolism syndrome” (PCS) [45]. Between the CRS and the PCS, the SARS-CoV-2 is playing havoc with our immune system. It remains to be seen whether the mast cells can be activated by autoantibodies in the absence of infection, which might be relevant to long COVID-19 with persistent symptoms [46]. The real question is whether this type of retrovirus and semi-retrovirus infections are going to make us a mass of life forms riddled with sick organs and cursed with self-mutilating autoimmune disorders.

**Figure 3:**
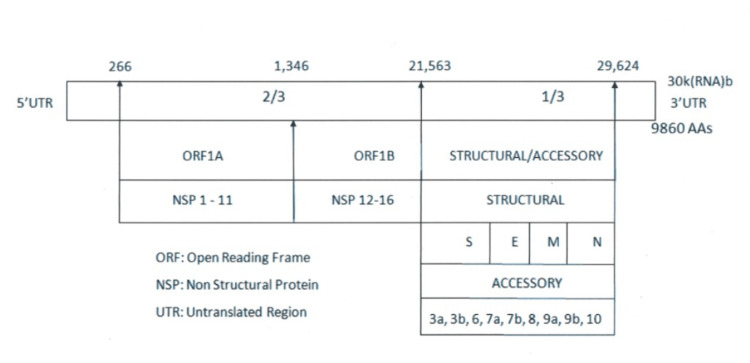
SARS-COV-2 genome Image credit: Madhavan Pisharodi, MD.

**Figure 4:**
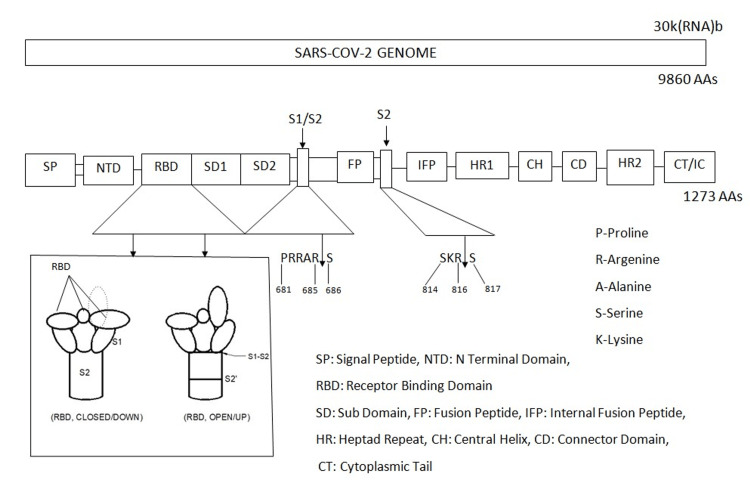
Genomes of SARS-CoV-2 and spike protein Image credit: Madhavan Pisharodi, MD.

We learned a lot about the special aspects of the structure and function of SARS-COV-2 that helped our efforts to control the infection. Information about the biological functions of the viral domains, including virus-host cell interaction, virus assembly, etc., can play an important role in the identification of regions that should be targeted during virus inactivation strategies [7]. SARS-CoV-2 is an enveloped, single-strand, positive sense RNA virus with a diameter of 60-140 nm and spikes of 9-12 nm in length [13]. It is part of the betacoronavirus genus, which includes Middle East respiratory syndrome-related coronavirus (MERS-CoV) and severe acute respiratory syndrome coronavirus (SARS-CoV) [47]. There are four structural proteins, nucleocapsid (N) with 419 amino acids inside the virions associated with RNA, and the spike (S), envelope (E), and membrane (M) proteins inserted into the lipidic viral envelope. The SARS-CoV-2 has 29-30k RNA bases that encode 9860 amino acids (Figure 3) [13]. The spike protein has about 1273 amino acids spread between the S1 and S2 segments [5,48-51]. The S1-S2 junction is between amino acids 685 and 686 and the S2 cleavage site is between amino acids 816 and 817 (Figure 4) [5,13].

There are many characteristics of the SARS-CoV-2 virus that make COVID-19 more dangerous than the previous coronavirus infections. Some of the significant structural and functional special features of SARS-CoV-2 and its RBD greatly contributed to the seriousness of the pandemic, and these are in addition to the infectivity of the virus in the asymptomatic and pre-symptomatic stages as well as its deceptively low morbidity and the low mortality rates. Increased affinity of RBD for ACE2, addition of S1S2 furin cleavage site (FCS), RBD masking, syncytium formation, preferential utilization of the cell surface entry using transmembrane protease serine subtype 2 (TMPRSS2), intracellular (infected cell) cleavage of S protein, preferential infection of the upper respiratory tract (URT), molecular mimicry, formation of autoantibody, and cytokine storm possibly through antibody-dependent disease enhancement (ADE), toll-like receptor-4 (TLR4) activation, and/or MCAS are some such special features this virus has utilized to create such a dangerous pandemic. These features made the control of COVID-19 very difficult and unpredictable during the peak months.

All these special features made SARS-CoV-2 in absolute control of infectivity, viral entry options, viral multiplication, pathogenicity, and evasion of host defenses. The pathogenesis of long COVID disease is also related to some of these special features of the SARS-CoV-2 virus. Effective control of the virus depends on our ability to prevent the viruses from getting into the human body.

Germicidal UVC

Ultraviolet irradiation (specifically UVC) deserves particular attention as one of the most effective antiviral strategies [7]. UVC capable of inactivating pathogenic microorganisms is usually called germicidal UVC or ultraviolet germicidal irradiation (UVGI).

The wavelength of UVC ranges from 200 to 280 nm and is not visible to the human eye. Ultraviolet A (UVA) (315-400 nm) and ultraviolet B (UVB) (280-315 nm) are also beyond the visible spectrum. The visible spectrum ranges from 400 to 700 nm. Infrared, microwave, and radio waves have longer wavelengths, and X-rays and gamma-rays have shorter wavelengths. Among the ultraviolet rays, UVA & UVB are mostly ineffective against SARS-CoV-2. UVA and UVB inactivate SARS-CoV-2, but have low efficiency compared to UVC [52,53]. Most ultraviolet rays are known to cause aging of the skin, wrinkling of the skin, and skin cancer, including melanomas [54]. They can also cause cataracts, weakening of the immune system, and stronger allergic reactions [55]. There is evidence that chronic exposure to high-intensity UVC light can lead to the development of age-related macular degeneration of the retina and cortical cataracts [56,57]. Prolonged and long-term exposure to UVC light can induce skin cancers, such as basal cell carcinomas, squamous cell carcinomas, and even malignant melanoma [58-60]. Because of the potential hazard due to the destructive and carcinogenic effects of UVC, the application of UVC light in public settings is a highly controversial issue [7]. In fact, for the purpose of protecting humans from the harmful effects of UVC light, the WHO put out ISO 15858 in 2016 to set standards, including the allowable maximum dosage per person. Through this unique device, the WHO restriction is eliminated because no UVC is coming out of the device. Sunscreen can protect us almost completely from UVA and UVB. UVC is filtered out by the naturally existing ozone layer in the stratosphere and prevents any UVC from coming down to the ground level. It is possible that microorganisms are destroyed by UVC because the microorganisms were not exposed to UVC due to the ozone layer filtration in the stratosphere. On the other hand, the microorganisms have developed “herd immunity” to UVA and UVB that are not filtered by ozone and freely exist at the ground level over millions or even billions of years in the past. Ozone layer filtration is considered to be protection for us. The ozone created in the lower atmosphere, i.e., the troposphere, by pollutants, and by UVC wavelength below 240, including Far UVC of 222, on the other hand, is considered harmful and can cause breathing disorders and damage to the respiratory tract. Troposphere ozone is also called ambient or ground-level ozone (EPA, USA).

UVC is easily created artificially by using low-pressure mercury fluorescent lamps. Our household fluorescent lamps produce short-wave UV inside when an electric current is passed through mercury vapor. This UV works on the phosphorus coating on the inside of the tube to produce the glow of fluorescent lamps that block UV and produce visible light. The UVC is blocked by the phosphor coating of the lamps that convert the UV light into higher frequency visible light. When creating UVC for germicidal use, some light waves near the UVC light waves (violet 380-450 and blue 450-485) leak out. This will make the UVC light come out as the so-called “blue light.” This leak does not affect the functions of UVC and not having to filter out the neighboring frequencies makes the process of creating UVC cheaper. Also, the visible “blue light” tells us that the invisible UVC is on or off.

The best germicidal UVC frequency is 262 nm (ranges from 250 to 270 nm) because at this frequency, the virus has the highest absorption potential resulting in the greatest photolysis leading to photodimerization of the nucleic acids (DNA and RNA) [61,62]. UVC can destroy viruses through several possible ways and different viruses are destroyed by different mechanisms. UVC can cause viral protein oxidation, as in some bacteriophages [63-65]. In some other viruses, UVC acts by destroying the capsid proteins [66]. In poliovirus, UVC causes virus protein-genome cross-linking [67]. UVC destroys the genome of influenza virus [68]. UVC damages the viral genome but not the viral proteins in adenoviruses and SARS-CoV-2 [69,70]. It is reasonable to assume that with larger doses of UVC, these multiple methods will overlap, and the damage will be more extensive. For instance, due to the limitation of the equipment, the authors could not conclude that UVC (at a higher dose) does not damage proteins of SARS-CoV-2 [70]. The dimerization leads to DNA/RNA disassembly resulting in disruption of cellular replication and other functions [71,72]. UVC light also denatures the enzymes needed for viral DNA/RNA repair [73]. As the wavelength gets shorter, the depth of penetration of the radiation goes down and the damage to RNA and DNA also goes down. However, this is compensated by increased damage to the proteins of the virus and other microorganisms. As a result, the UVC frequencies of 207-222, known as Far UVC, behave differently from the UVC of 253.7 nm. For instance, a Far UVC of 222 nm is known to be less harmful to the skin and more lethal to the virus through a combination of protein damage and some amount of RNA and DNA damage. UVC 222 nm has recently shown potential for SARS-CoV-2 disinfection [7]. Any UVC below the frequency of 240 nm creates ozone as a byproduct. As a result, the Far UVC of 222 nm carries a new problem of having to filter out the ozone, not associated with the UVC of 253.7 nm [74].

Making UVC safe and effective for total protection

**Figure 5:**
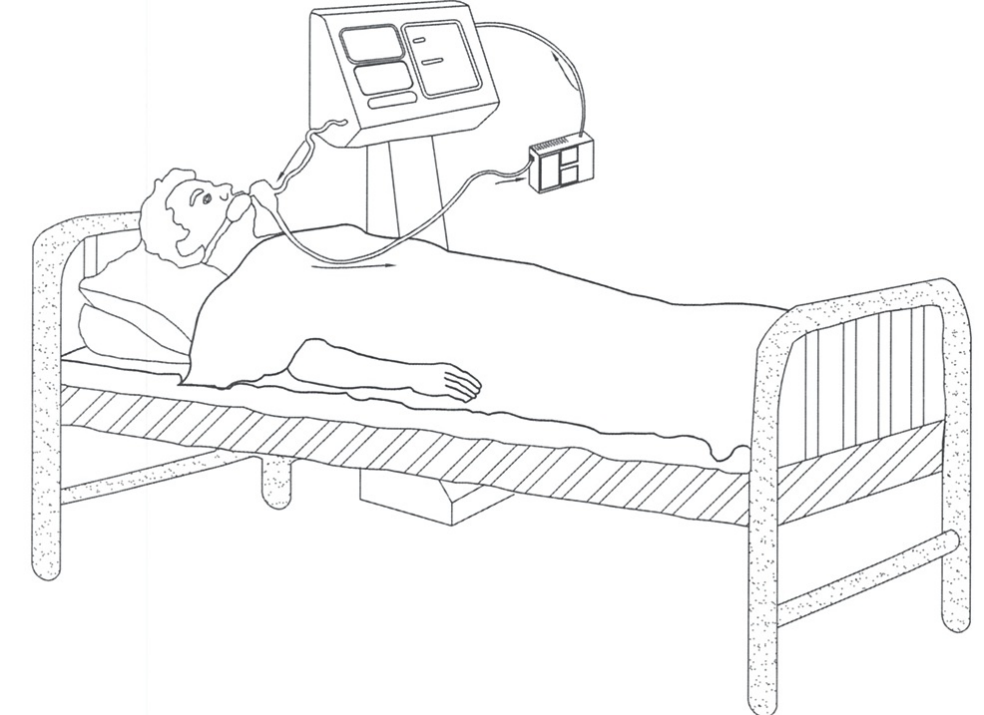
Protection of a person on a ventilator Image credit: Madhavan Pisharodi, MD.

**Figure 6:**
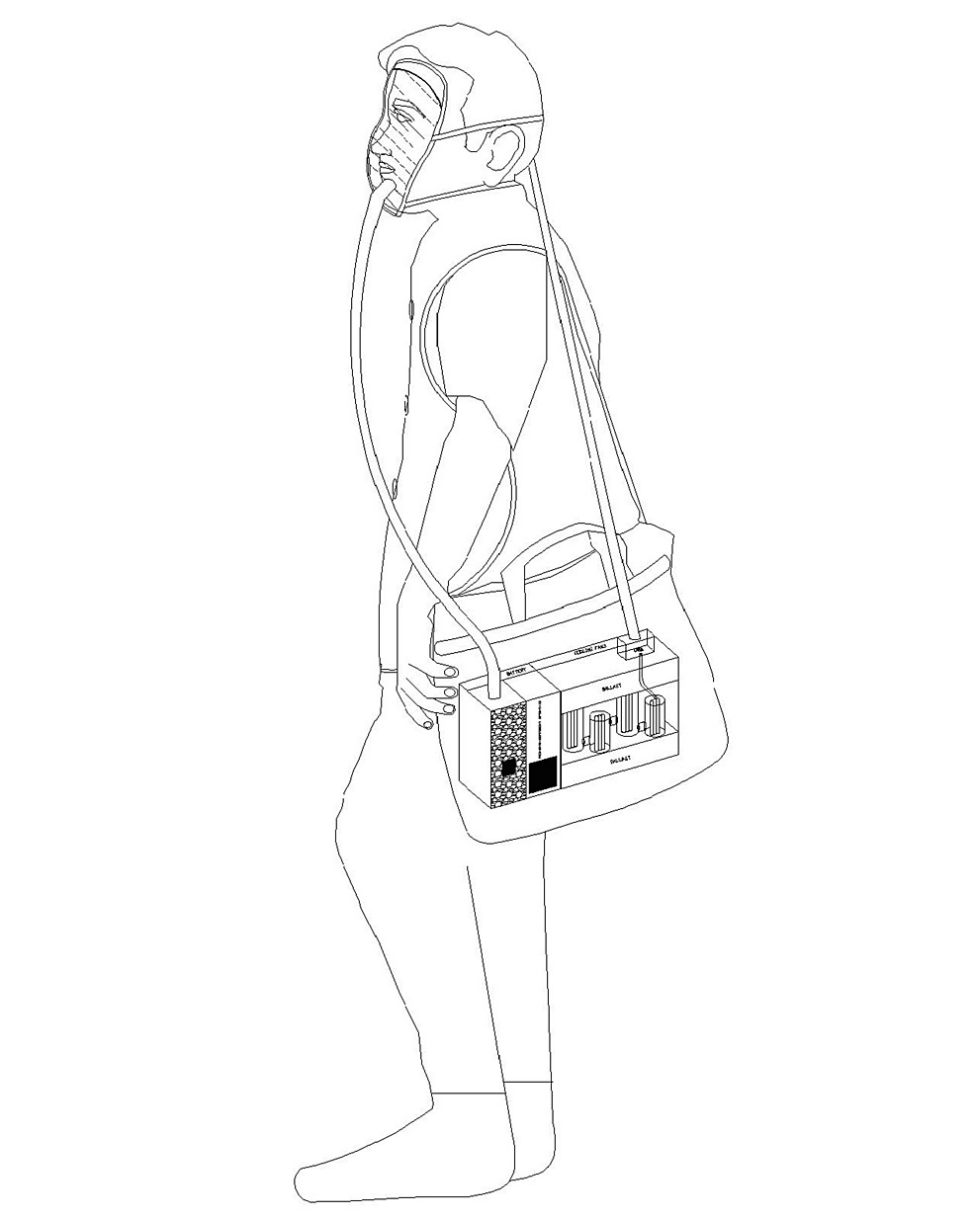
One way to use the portable, personal bio-protection device (PPBD) Image credit: Madhavan Pisharodi, MD.

The conventional recommendation is not to use UVC on live animals due to safety concerns [70]. This is not stating that ultraviolet disinfection is not already in use. The PPBD is a novel concept that can overcome almost all of the limitations of currently available UVC systems. UVC is being used with some precautionary measures to inactivate SARS-CoV-2 in different surface materials [52,75,76]. Also, UVC is being used in healthcare facilities for environmental disinfection [77]. This negative feeling against UVC can be reversed with some modifications. In our efforts to make UVC 253.7 nm user-friendly, two of its major problems are addressed in our two units presented here through a unique and quite novel technique. The first major problem is the damage to the skin and eyes. Our units deal with this problem by achieving virus inactivation inside the UVC chambers enclosed in UVC opaque housings remote from the end user. This ensures that no UVC is coming out and the UVC is not in direct contact with the end user at any time. The second major disadvantage of any UVC device is that light travels only in straight lines (except in the space-time models) and the objects in the light pathways will create shadows and allow the microorganisms to hide behind such shadows. Our device uniquely empowers the air to mop up the microorganisms from all the nooks and corners and to bring them to the UVC chambers for inactivation. We have tested one unit for individual usage. A closed-circuit system is created such that the individual can breathe exclusively the disinfected air through a mask or a ventilator (Figures 5, 6). For the buildings, there is a way to create self-cleaning air circulation and this air, loaded with microorganisms, is passed through larger UVC units arranged in line with the airflow where the AC air cannot go around the unit and has to pass through the unit before allowing the air back into the different sections of the buildings. Using this method, the air coming back into the living areas is always disinfected and purified. A six-chamber unit was tested and found to be effective for this purpose. Multiple serial units may be needed for larger AC units.

**Figure 7:**
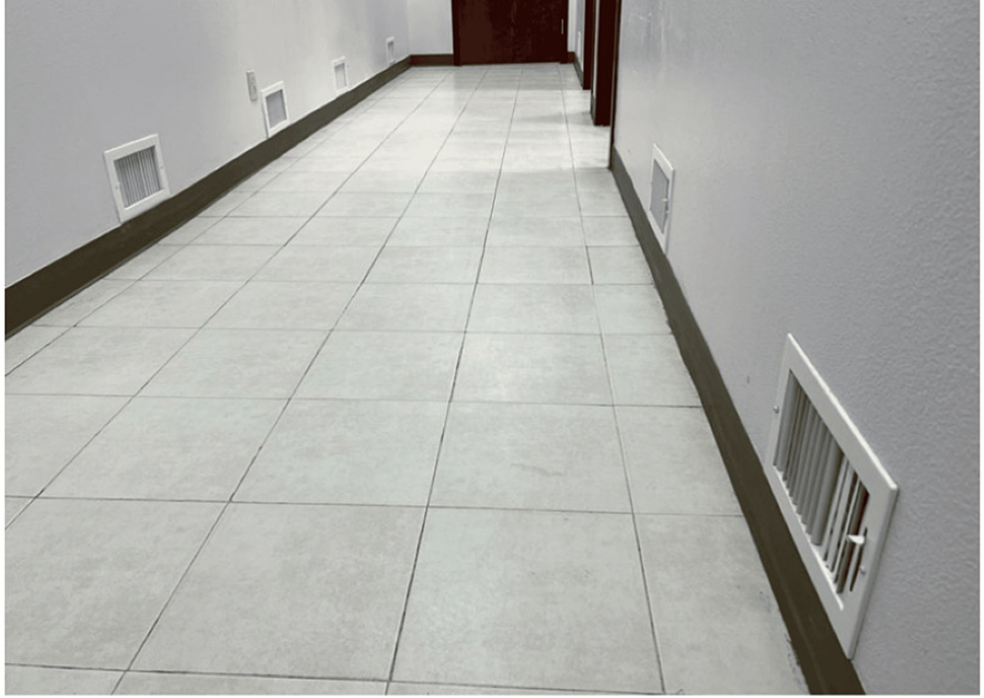
Vents at ground level in our clinic Image credit: Madhavan Pisharodi, MD.

**Figure 8:**
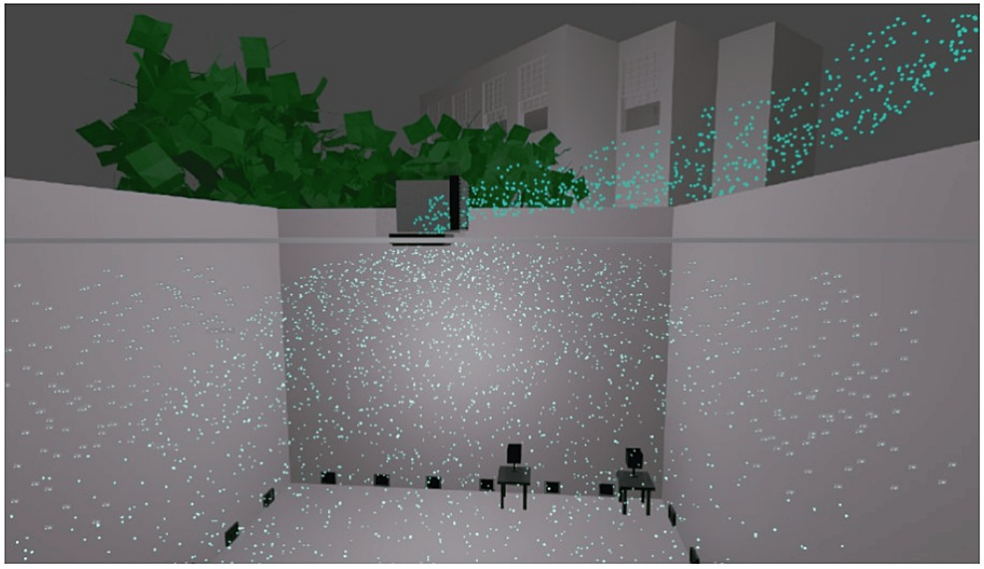
The air being discarded to the outdoors Image credit: Madhavan Pisharodi, MD.

**Figure 9:**
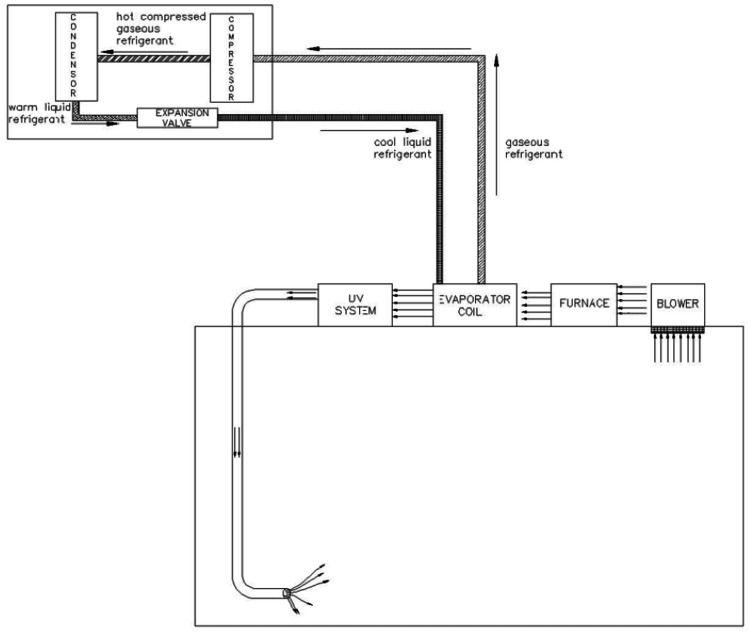
The contaminated air passes through the disinfection chamber unit and recirculates into the room UV: ultraviolet. Image credit: Madhavan Pisharodi, MD.

Most of the present AC units provide the air from the ceiling down and the air from the room goes back to the AC units also through the ceiling. This creates stronger circulation in the upper segments of the room, especially in cold climates because the warm air always goes to the top. The cooler, slower air with low humidity due to the air conditioning, in the lower segments of the room, allows the viruses to stay active there for longer periods. In a self-cleaning system, the air is made to come out at ground level (Figure 7). The air will then carry the microorganisms in their currents to exit through the ceiling (Figure 8). This contaminated air going out through the ceiling is either discarded into the air above the buildings or recirculated after passing through disinfection chamber units enclosed in UVC-opaque housings and located in the attic inside the air ducts (Figure 9). Either way, the air going back into the room is totally free of microorganisms, and the individuals in the room are not exposed to any UVC. This system is unique and novel and is covered through patents [24-26] and publications [27,28].

Contactless passive immunization

A method of immunizing individuals without having to administer the antigen through injections, oral, or other applications is considered here. This method has the additional benefit of becoming available at the start of the outbreak, before the other forms of vaccines are developed, and even before the pandemic is established. This novel concept has unlimited potential. Additionally, the delay between the starting of a new variant and the creation of the new vaccine can be greatly reduced.

COVID-19 is turning out to be a chronic viral menace due to the new variants that mutate S protein to achieve immune evasion. Unfortunately, SARS-CoV-2 variants may continue to adapt in the human population for years or decades. The current vaccine strategies focused against S protein may soon become impractical [5]. Vaccines targeting various SARS-CoV-2 proteins are under development [78]. A polyvalent vaccine with more than just the S protein may provide better and/or longer protection. CORONA VAC (China) and COVAXIN (India) are two such vaccines. These vaccines did not perform to the level expected of them. There is a possibility that the quality of antigens was compromised by the method of inactivation of the virus. These inactivating methods behave like cluster bombs on the virus. UVC is capable of inactivating the virus, behaving like a strategic precision bomb. Multiple studies have shown that the UVC in the appropriate dosage can inactivate the virus without altering its architecture or destroying the proteins. The viral particle morphology remained intact even when the virus completely lost infectivity after UVC radiation [66]. The results provided evidence that UVC inactivates SARS-CoV-2 by inducing viral genome damage [70]. Even the genome damage is very precise with the photodimerization of a few nucleotypes, just enough to make the nucleic acid unable to replicate. This supports the possibility that SARS-COV-2 viral morphology and proteins are not severely damaged after UVC treatment [70]. Also, the UV-inactivated SARS-CoV vaccine has been shown to have the ability to elicit systemic humoral immunity against viral spike and nucleocapsid protein in mice [79]. If ACs can provide such inactivated viruses with intact antigenic proteins to these facilities, then the individuals in such facilities may be getting passive immunity by breathing in the inactivated virus particles with the four antigenic proteins. Since cells in the nose contain ACE2 protein, it seems reasonable to conclude that the primary infection and virus replication take place in the nose [3]. The inactivated multi-antigen virus can immunize starting through the initial landing site of the SARS-CoV-2. Such multi-antigen-induced immunity will be better than single antigen (S protein) induced protection and will bring the level of vaccine-induced protection to that of natural immunity after actual infection. It is even possible that the spikes from the inactivated virus particles with intact morphology can competitively block the angiotensin-converting enzyme (ACE) proteins from accepting active virus particles, for a brief period. The feasibility of movable immunization chambers is also unique and something to be considered. This can be in the form of mobile homes. The stored virus is fed into these mobile home-like facilities and destroyed by passing through UVC chambers and the resulting neutered virus is released into the vehicle where the individuals can be immunized passively by staying there and breathing the antigen-loaded air. Such a contactless passive immunization plan may have application in livestock infections also.

## Conclusions

The two units presented here were already tested and shown to disinfect the air totally for human breathing. The small unit is for individual use. The large unit or a modified version of it can be used for community protection. Such a unit can be added to a self-cleaning air-flow system incorporated into the AC and can be installed in buildings, public transportation units, and any other places where people gather around. These units can reduce the complications of having to fight the virus inside the human body. By treating the air remotely in a UVC-opaque housing, the end user is not exposed to the UVC radiation. The system also prevents the problem of viruses hiding in the shadow created in the way of the light rays. This will make such units practical, reliable, acceptable, affordable, and safe for individual use and also for use in airplanes, trains, ships, etc., in addition to the buildings. The real importance is that the microorganisms are stopped in the air and prevented from entering the human body, thus avoiding acute and long COVID-like problems.

The individual protection is provided through a single or two-chamber unit (PPBD) that is connected to a transparent air-tight face mask through a tubing. A similar unit can be used for the ventilator also. For the ACs, larger units, incorporated into the AC duct, will be needed. The AC system units will disinfect and purify the air, and recirculate it through the air conditioner; this can be used to protect facilities like acute and long-term healthcare facilities and other buildings, airplanes, trains, buses, ships, etc., where people gather in large numbers. Health care is provided by individuals in specialized facilities. A system that protects these individuals and these facilities indirectly protects the general population. We should be able to prevent another pandemic by zooming down on any such outbreaks and limiting its spread. The PPBD and the modified AC with disinfection have great potential to help in this effort. Additional benefits in possibly reducing HAI is a major collateral benefit.
